# Storage Period and Different Abiotic Factors Regulate Seed Germination of Two *Apocynum* Species — Cash Crops in Arid Saline Regions in the Northwestern China

**DOI:** 10.3389/fpls.2021.671157

**Published:** 2021-06-17

**Authors:** Li Jiang, Chaowen She, Changyan Tian, Mohsin Tanveer, Lei Wang

**Affiliations:** ^1^Key Laboratory of Research and Utilization of Ethnomedicinal Plant Resources of Hunan Province, Huaihua University, Huaihua, China; ^2^Key Laboratory of Hunan Higher Education for Western Hunan Medicinal Plant and Ethnobotany, Huaihua University, Huaihua, China; ^3^State Key Laboratory of Desert and Oasis Ecology, Xinjiang Institute of Ecology and Geography, Chinese Academy of Sciences, Ürümqi, China; ^4^University of Chinese Academy of Sciences, Beijing, China; ^5^Tasmanian Institute of Agriculture, University of Tasmania, Hobart, TAS, Australia

**Keywords:** *Apocynum*, drought, salinity, seed germination, seed storage

## Abstract

On degraded land in arid regions, cultivation of *Apocynum* species can provide significant environmental benefits by preventing soil erosion and desertification. Furthermore, *Apocynum venetum* and *Apocynum pictum*, which are mainly distributed in salt-barren lands in the northwestern region of China, are traditionally used to produce natural fiber and herbal tea. Direct sowing of both species may encounter various abiotic stresses such as drought and salinity. However, these effects on germination remain largely unknown, especially for seeds with different storage periods. The aim of this study was to evaluate the effects of storage period, light condition, temperature regime, drought, and salinity on germination performances of both species. Germination experiment was carried out in November 2017. There were four replicates for each treatment, and each petri dish contained 25 seeds. The results indicated that prolongation of storage period significantly decreased the germination percentage and velocity, especially under abiotic stresses. Light did not affect seed germination of *A. venetum* and *A. pictum* under any conditions. Seeds had better germination performance at 10/25 and 15/30°C than those of seeds incubated at any other temperatures. With the increase of polyethylene glycol (PEG) and salinity concentrations, seed germination for both species gradually decreased, especially for seeds stored for 2 years. Low PEG (0–20%) and salinity concentration (0–200 mM) did not significantly affect germination percentage of freshly matured seeds. However, long-time storage significantly decreased drought and salinity tolerance in *A. venetum* and *A. pictum* during germination stage. For saline soils in arid and semi-arid regions, freshly matured seeds or 1-year-stored seeds of both *Apocynum* species are recommended to be sown by using drip-irrigation in spring.

## Introduction

Land degradation, caused by inappropriate land uses and climate change, is considered as the most threaten environmental issue, especially in arid or semi-arid regions (Mao et al., 2018). In Central Asia, land degradation and the decrease of productivity significantly influence the ecological, social, and economic sustainability of ecosystems. To restore the degraded land in this area, revegetation of native herb perennial is a proper method (Lopez et al., 2017). *Apocynum* species are suitable plants for restoration because they are easy to propagate and have the ability to withstand the harsh desert environment. *Apocynum venetum* and *Apocynum pictum* are perennial halophytic herbs belonging to the Apocynaceae family (Thevs et al., 2012; Xie et al., 2012). Cultivation of both species can provide significant environmental benefits by preventing land degradation caused by salinization and desertification and offer opportunities to develop desert farming in the arid zones (Ping et al., 2014; Rouzi et al., 2018).

Due to its physical, structural, mechanical, and antibacterial properties, the phloem fiber obtained from the inner bark of both *Apocynum* species is used to make strings, fishing nests, and high-quality paper, especially used in textile industry to produce comfortable and healthy fabrics and clothing (Han et al., 2008; Xu et al., 2020). Far infrared clothing made of *Apocynum* fiber can improve blood circulation and stabilize blood pressure (Xie et al., 2012). Leaves of both species can yield up to 5% of gum, which is used to produce rubber (Flora of China Editorial Committee., 2006). Moreover, their leaves are commonly used to make luobuma tea and traditionally used as a sedative, as well as in the treatment of hypertension (Xie et al., 2012; Jiang et al., 2019).

Seed germination is the most critical transition period for crop establishment, especially under adverse abiotic conditions (Campobenedetto et al., 2020). Successful establishment of seedlings largely depends on timely germination and uniform emergence under different abiotic stresses such as drought and salinity (Rasheed et al., 2019). Although studies focusing on germination have been undertaken for *A. venetum* and *A. pictum* seeds, the majority of investigations have used freshly matured seeds and under a single environmental factor (Shi et al., 2014; Liu et al., 2015; Xu et al., 2015). No comprehensive information is available regarding germination of stored *Apocynum* seeds to various abiotic stresses. A better understanding of seed storage and abiotic factors affecting germination can help to optimize cultivation condition of both *Apocynum* species. Furthermore, testing the stress tolerance ability of *Apocynum* seeds will give us useful guideline for their sustainable cultivation.

There are differences among species in seed germinability in responding to storage, even among species of the same genus (Bettey et al., 2000; Li et al., 2007). For instance, *Mesembryanthemum nodiflorum* seeds can germinate after stored dry at ambient laboratory temperatures for 32 years (Gutterman and Gendler, 2005). However, after 3 months of storage, germination percentage of *Tamarix parviflora* seeds decreases from 89 to 8%, and from 70.6 to 0% for *Tamarix gallica* (Terrones et al., 2016). Seed storage affects germination performance and seed viability depending on the period of time and conditions of storage (Panobianco et al., 2007; Lozano-Isla et al., 2018). During storage, orthodox seeds gradually lose their viability mainly caused by the accumulation of reactive oxygen species (ROS), which increase lipid oxidation and hydrolysis (López-Fernández et al., 2018; Wiebach et al., 2019).

Delayed and poor germination performance can negatively affect crop growth and yield (Willenborg et al., 2005). The abiotic stresses that might restrict seed germination of *A. venetum* and *A. pictum* include unsuitable light condition, extreme temperature, drought, and salinity. It is relatively easy to regulate light and temperature condition via adjustment of planting depth and selection of suitable sowing date in agriculture management. For seed germination of industrial crops in arid and semi-arid regions, drought and salinity stresses are the main limitation factors (Rasheed et al., 2019). Drought and salinity can delay or completely inhibit germination (Zhu et al., 2006; Toscano et al., 2017; Hu et al., 2018; Tang et al., 2019). Seeds must first imbibe an adequate amount of water to start germination process, which depends on the osmotic pressure of soil (Wuest, 2007). However, drought and/or salinity increase osmotic pressure and thus impair seed imbibition and seed germination (Lin et al., 2016). Freshly matured seeds of *A. venetum* can germinate at water potential values from −0.6 to −0.90 MPa and at 0–200 mM salinity concentration (Rong et al., 2015; Xu et al., 2015). Thus, freshly matured seeds of *A. venetum* exhibit high tolerance to osmotic stresses. However, we do not know germination performance of *A. venetum* seeds stored for different periods under different drought and salinity levels.

*Apocynum venetum* and *A. pictum* are perennial plants and adapt to arid climate. *A. venetum* is an inhabitant of Europe and Southwest Asia, and in China, it is mainly distributed in the arid region of the northwestern China (Xie et al., 2012). *A. pictum* is distributed in southern Kazakhstan, northwestern China, and Mongolia and is restricted to the arid zones (Thevs et al., 2012). The habitats for *A. venetum* and *A. pictum* are salt-barren zone, desert margins, and riversides. However, no comparative germination studies have been conducted on both *Apocynum* species. Based on the differences in their ecological distribution, we hypothesized that seeds of both species might have different responses to temperature and drought. The objective of this study was to investigate the recommended storage period for optimum germination performance of both *Apocynum* species in response to light, temperature, drought, and salinity.

## Materials and Methods

### Seeds

Freshly matured fruits of *A. venetum* and *A. pictum* were collected during three consecutive autumns, 2015, 2016, and 2017, from cultivated plants at National Fukang Desert Ecosystem Field Sciences Observation and Research Station, Chinese Academy of Sciences (44°17’N, 87°56’E), Xinjiang, China. Fruits were collected from more than 100 plants for each species. After naturally dry, seeds were detached from fruits and then stored in room temperature (22 ± 3°C) and dry conditions in darkness.

### Germination Tests

Germination experiment was carried out in November 2017. There were four replicates for each treatment, and each petri dish contained 25 seeds. Seeds were randomly placed in 5-cm-diameter Petri dishes on two layers of filter paper and moistened with 2.5 ml of test solutions. Petri dishes were sealed with parafilm. Embryo protrusion ≥ 1 mm was the criterion for seed germination. Germination in light (12-h darkness/12-h light photoperiod) was examined daily for 15 days, and germinated seeds were removed.

#### Species, Storage Time, Light, and Temperature

A 2 × 3 × 2 × 5 factorial design was used to assess the effects of species, storage time, light, and temperature. To determine the effects of temperature on germination, 12-h alternating temperature regimes of 5/15, 5/20, 10/25, 15/30, and 20/35°C were used. Freshly matured seeds of both *Apocynum* species or seeds stored for 1 or 2 years were germinated in distilled water at the above-mentioned temperature regimes in light and in continuous darkness (petri dishes wrapped with two layers of aluminum foil). Seeds incubated in darkness were checked at the end of the experiment.

#### Species, Storage Time, and Drought

A 2 × 3 × 8 factorial design was used to assess the effects of species, storage period, and drought. Freshly matured seeds or seeds stored for 1 or 2 years were germinated in distilled water or in 5, 10, 15, 20, 25, 30, and 40% polyethylene glycol (PEG-6000) at 10/25°C.

#### Species, Storage Time, and Salinity

A 2 × 3 × 7 factorial design was used to assess the effects of species, storage period, and salinity. Freshly matured seeds or seeds stored for 1 or 2 years were germinated in distilled water or in 50, 100, 200, 300, 400, and 600 mM NaCl at 10/25°C.

### Statistical Analyses

The velocity of germination was calculated using a modified Timson’s index (Khan and Ungar, 1984). Because germination data did not meet the assumptions of three-way and four-way ANOVA, data were analyzed by linear regression. For germination test of Section “Species, Storage Time, Light, and Temperature,” the multiple linear regression model included plant species, storage period, light, and temperature. For germination test of Section “Species, Storage Time, and Drought,” the multiple linear regression model included plant species, storage period, and PEG concentration. For germination test of Section “Species, Storage Time, and Salinity,” the multiple linear regression model included plant species, storage time, and salinity. Tukey’s test (*P* < 0.05) was carried out to determine whether significant differences occurred between individual treatments.

## Results

### Species, Storage Time, Light, and Temperature

**FIGURE 1:**
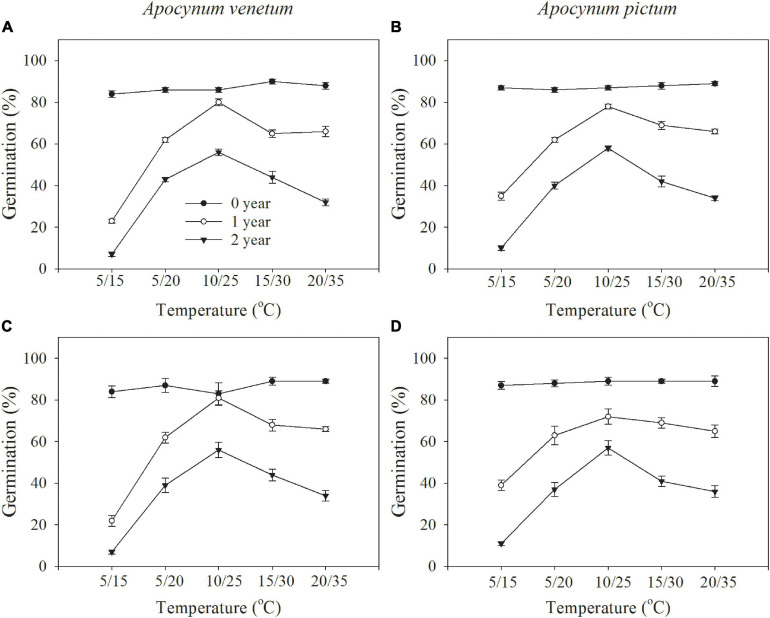
Germination responses of *Apocynum venetum* and *Apocynum pictum* seeds stored for 0, 1, and 2 years to 12-h darkness/12-h light **(A,B)** or 24-h darkness **(C,D)** under different temperatures.

Germination percentage was significantly affected by plant species (*P* < 0.05), storage period (*P* < 0.001), and temperature (*P* < 0.01). Light condition did not significantly affect seed germination for both *Apocynum* species under all test temperature regimes (Figure 1). Because germination was checked at the end of the experiment in continuous darkness, germination index was just calculated in light treatment. Germination index was significantly affected by plant species (*P* < 0.01), storage time (*P* < 0.001), and temperature (*P* < 0.001).

**FIGURE 2:**
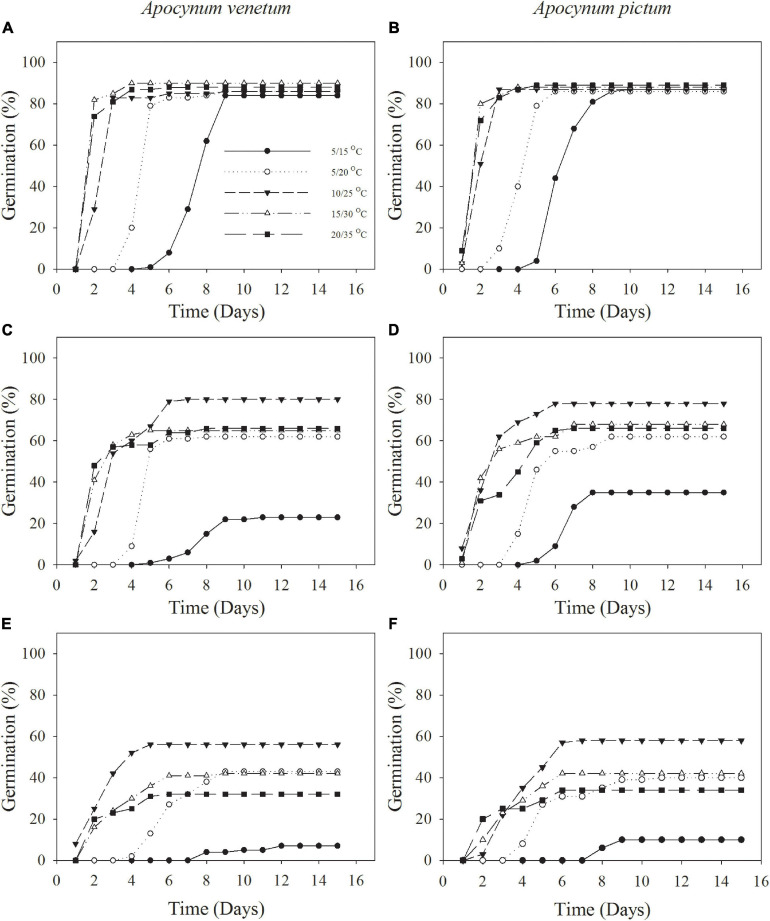
Cumulative germination percentages of *Apocynum venetum* and *Apocynum pictum* seeds stored for 0 year **(A,B)**, 1 year **(C,D)**, and 2 years **(E,F)** under different temperatures.

Freshly matured seeds or seeds stored for 2 years, both *Apocynum* species did not show significant difference in germination percentage. The difference between *A. venetum* and *A. pictum* seeds exited in 1-year storage treatment. At this treatment, *A. venetum* seeds had higher germination percentage than that of *A. pictum* at 10/25°C. With the increase of storage time, germination percentage and germination index were decreased dramatically (Figure 2 and Table 1). Overall, for all storage periods, germination performance was optimum at 10/25 and 15/30°C, and worst at 5/15°C.

**TABLE 1:** Effect of temperature on germination index of *Apocynum venetum* and *Apocynum pictum* seeds stored for 0, 1, or 2 years.

**Temperature (°C)**	***Apocynum venetum***			***Apocynum pictum***		
	**0**	**1**	**2**	**0**	**1**	**2**
5/15	45.9 ± 0.9dA	12.3 ± 0.3dB	3.1 ± 0.3dC	53.7 ± 1.0dA	21.2 ± 1.4eB	5.1 ± 0.5dC
5/20	63.4 ± 0.7cA	45.5 ± 0.8cB	27.5 ± 0.6cC	65.9 ± 0.6cA	44.1 ± 0.8dB	27.3 ± 0.9cC
10/25	75.7 ± 1.2bA	66.5 ± 1.4aB	45.8 ± 0.8aC	79.0 ± 0.9bA	68.5 ± 1.5aB	45.6 ± 1.1aC
15/30	83.1 ± 1.0aA	58.5 ± 1.9bB	35.2 ± 1.9bC	81.5 ± 1.1aA	60.1 ± 1.3bB	34.5 ± 1.8bC
20/35	80.6 ± 1.4aA	58.5 ± 1.9bB	27.9 ± 1.5cC	82.0 ± 0.4aA	55.4 ± 1.0cB	29.3 ± 1.0cC

*Different upper-case letters indicate significant difference between 0, 1, and 2 years at the same concentrations; different lower-case letters indicate significant difference temperature at *P* < 0.05.*

### Species, Storage Time, and Drought

**FIGURE 3:**
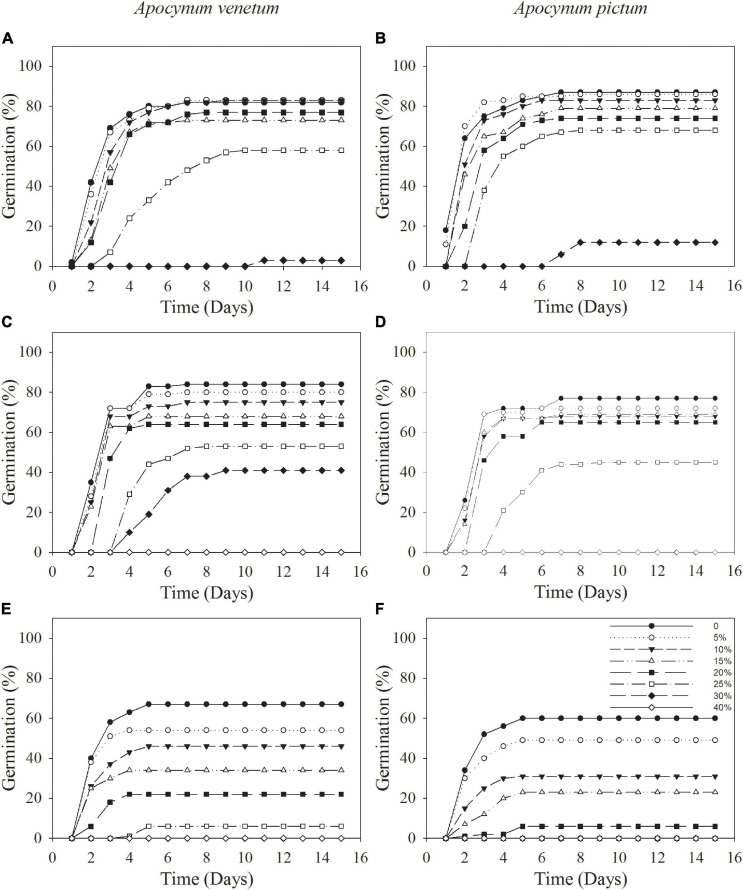
Cumulative germination percentages of *Apocynum venetum* and *Apocynum pictum* seeds stored for 0 year **(A,B)**, 1 year **(C,D)**, and 2 years **(E,F)** under different PEG concentrations.

Germination percentage and index were significantly affected by plant species (*P* < 0.05), storage period (*P* < 0.001), and PEG concentration (*P* < 0.001). Stored seeds of *A. venetum* had higher germination percentage and velocity than that of *A. pictum* under high PEG concentration. For example, germination percentage of *A. venetum* seeds stored for 1 year was 41 ± 9% under 30% PEG, whereas that of *A. pictum* seeds was 0. For seeds of *A. venetum* and *A. pictum*, the longer the storage period was, the lower the germination percentage and velocity were. With the increase of PEG concentration, performance of both species gradually decreased, especially for seeds stored for 2 years (Figure 3 and Table 2). Low PEG concentration (0–20%) did not significantly affect germination percentage of freshly matured seeds.

**TABLE 2:** Effect of PEG on germination index of *Apocynum venetum* and *Apocynum pictum* seeds stored for 0, 1, or 2 years.

**PEG (%)**	***Apocynum venetum***			***Apocynum pictum***		
	**0**	**1**	**2**	**0**	**1**	**2**
0	73.5 ± 3.1Aa	73.4 ± 1.4Aa	59.9 ± 1.3Ba	72.1 ± 0.8Aa	66.9 ± 0.8Ba	53.5 ± 1.4Ca
5	76.8 ± 2.1Aa	70.0 ± 2.2Ba	49.1 ± 0.9Cb	68.7 ± 0.9Aa	63.4 ± 4.5Aa	43.7 ± 0.8Bb
10	73.4 ± 2.0Aa	65.5 ± 2.4Bab	40.8 ± 0.9Cc	67.4 ± 2.5Aa	59.1 ± 3.1Aab	27.4 ± 1.6Bc
15	69.3 ± 2.1Aa	59.8 ± 6.9Aab	30.9 ± 2.2Bd	63.7 ± 1.5Aa	59.7 ± 4.6Aab	19.5 ± 0.9Bd
20	66.2 ± 6.7Aab	54.2 ± 2.3Ab	19.2 ± 1.7Be	63.9 ± 2.9Aa	54.1 ± 3.9Ab	4.7 ± 4.2Be
25	62.3 ± 3.2Ab	40.2 ± 6.3Bc	4.5 ± 1.9Cf	46.7 ± 4.3Ab	33.0 ± 5.0Bc	–
30	28.2 ± 3.0Ac	28.2 ± 7.5Ad	–	23.9 ± 2.1c	–	–
40	–	–	–	–	–	–

*Different upper-case letters indicate significant difference between 0, 1, and 2 years at the same concentrations; different lower-case letters indicate significant difference among different concentrations of PEG at *P* < 0.05.*

**FIGURE 4:**
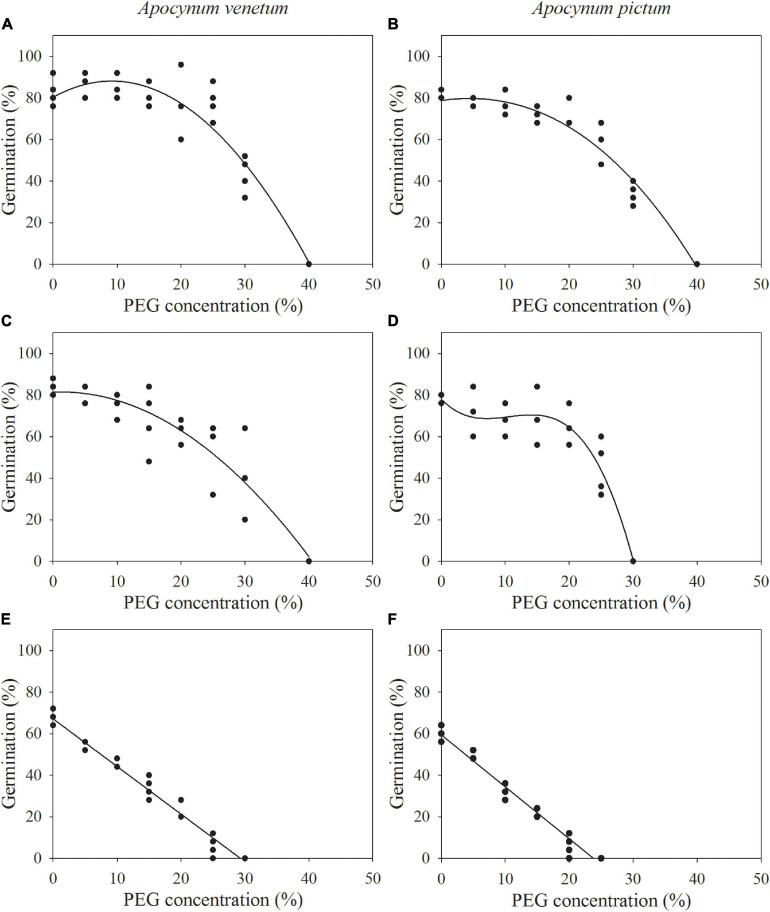
Relationship between germination percentages of *Apocynum venetum* and *Apocynum pictum* seeds stored for 0 year **(A,B)**, 1 year **(C,D)**, and 2 year **(E,F)** and PEG concentrations.

The regression equation of the relationship between PEG concentrations and germination percentages of freshly matured seeds of *A. venetum* was *y* = −0.0918*x*^2^ + 1.6845*x* + 80.4314 (*R*^2^ = 91%). The simulated critical value of PEG concentration for freshly matured *A. venetum* seeds (when germination percentage is 50%) was 29.56%, and the simulated limit value (when germination percentage is 0%) was 40.16%. For seeds stored for 1 year, the equation was *y* = −0.0528*x*^2^ + 0.1336*x* + 81.4174 (*R*^2^ = 88%), simulated critical value was 25.69%, and simulated limit value was 40.55%. For *A. venetum* seeds stored for 2 years, *y* = −2.2929*x* + 67.1071 (*R*^2^ = 97%), simulated critical value was 7.46%, and simulated limit value was 29.70% (Figure 4).

For freshly matured seeds of *A. pictum*, the equation was *y* = −0.0.0685*x*^2^ + 0.7662*x* + 78.0434 (*R*^2^ = 95%), simulated critical value was 26.58%, and simulated limit value was 39.81%. For *A. pictum* seeds stored for 1 year, the equation was *y* = −0.0104*x*^3^ + 0.3319*x*^2^ − 3.1365*x* + 78.0000 (*R*^2^ = 92%), simulated critical value was 24.02%, and simulated limit value was 30.16%. For *A. pictum* seeds stored for 2 years, *y* = −2.4971*x* + 59.3810 (*R*^2^ = 97%), simulated critical value was 3.76%, and simulated limit value was 23.78% (Figure 4).

### Species, Storage Time, and Salinity

**FIGURE 5:**
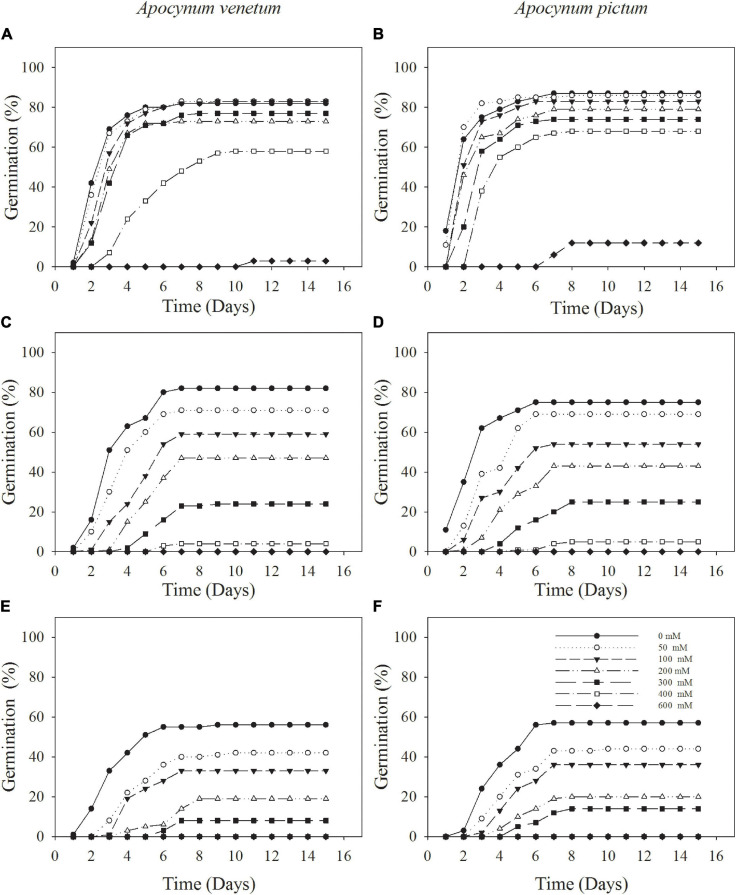
Cumulative germination percentages of *Apocynum venetum* and *Apocynum pictum* seeds stored for 0 year **(A,B)**, 1 year **(C,D)**, and 2 years **(E,F)** under different NaCl concentrations.

Germination percentage and index were significantly affected by plant species (*P* < 0.05), storage time (*P* < 0.001), and salinity (*P* < 0.001). Freshly matured and 1-year storage seeds of *A. pictum* and *A. venetum* had similar germination percentage and velocity (Figure 5 and Table 3). For example, germination percentage of *A. venetum* seeds stored for 1 year was 59% under 100 mM salinity, and that of *A. pictum* seeds was 54%. For both species, germination percentage and velocity decreased with the increase of storage period. Germination of both species gradually decreased with the increase of salinity. Low salinity concentration (0–200 mM) did not significantly affect germination percentage of freshly matured seeds.

**TABLE 3:** Effect of salinity on germination index of *Apocynum venetum* and *Apocynum pictum* seeds stored for 0, 1, or 2 years.

**NaCl**	***Apocynum venetum***			***Apocynum pictum***		
**(mmol L^–1^)**	**0**	**1**	**2**	**0**	**1**	**2**
0	72.5 ± 4.7Aa	67.8 ± 1.6Ba	46.5 ± 1.7Ca	79.1 ± 3.0Aa	66.4 ± 0.7Ba	45.1 ± 1.5Ca
50	72.1 ± 3.5Aab	57.3 ± 0.8Bb	31.1 ± 1.1Cb	79.3 ± 2.3Aa	56.4 ± 1.4Bb	32.5 ± 1.1Cb
100	70.2 ± 3.5Aab	44.2 ± 0.5Bc	24.6 ± 1.5Cc	74.0 ± 3.2Aab	42.9 ± 1.5Bc	26.1 ± 1.4Cc
200	62.0 ± 4.2Aab	33.4 ± 0.8Bd	12.0 ± 0.6Cd	69.3 ± 3.7Aab	31.9 ± 1.5Bd	13.8 ± 1.5Cd
300	58.9 ± 5.8Ab	16.1 ± 1.4Be	5.0 ± 1.1Ce	63.5 ± 2.3Ab	16.8 ± 1.2Be	9.1 ± 0.6Ce
400	36.9 ± 4.9Ac	2.6 ± 0.1Bf	–	55.3 ± 3.1Ac	3.1 ± 0.8Bf	–
600	1.0 ± 0.6d	–	–	6.8 ± 1.0d	–	–

*Different upper-case letters indicate significant difference between 0, 1, and 2 years at the same concentrations; different lower-case letters indicate significant difference among different concentrations of salinity at *P* < 0.05.*

**FIGURE 6:**
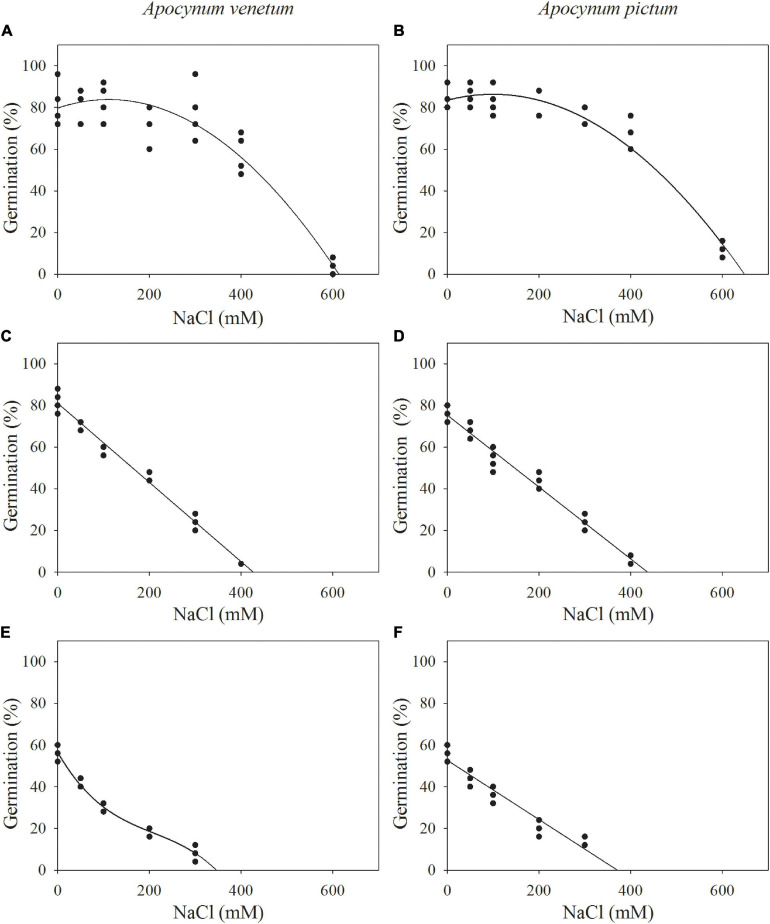
Relationship between germination percentages of *Apocynum venetum* and *Apocynum pictum* seeds stored for 0 year **(A,B)**, 1 year **(C,D)**, and 2 years **(E,F)** and NaCl concentrations.

The regression equation of the relationship between salinity concentrations and germination percentages of freshly matured *A. venetum* seeds was *y* = −0.0003*x*^2^ + 0.0738*x* + 79.7764 (*R*^2^ = 89%). The simulated critical value of salinity concentration for freshly matured *A. venetum* seeds was 431 mM and the simulated limit value was 613 mM. For *A. venetum* seeds stored for 1 year, the equation was *y* = −0.1462*x* + 75.4648 (*R*^2^ = 93%), simulated critical value was 174 mM, and simulated limit value was 516 mM. For *A. venetum* seeds stored for 2 years, *y* = −2.2281E-6*x*^3^ + 0.0014*x*^2^ −0.3789*x* + 56.4135 (*R*^2^ = 98%), simulated critical value was 19 mM, and simulated limit value was 349 mM (Figure 6).

For freshly matured *A. pictum* seeds, the equation was *y* = −0.0003*x*^2^ + 0.0573*x* + 83.5292 (*R*^2^ = 95%), simulated critical value was 456 mM, and simulated limit value was 649 mM. For *A. pictum* seeds stored for 1 year, the equation was *y* = −0.1724*x* + 75.3404 (*R*^2^ = 97%), simulated critical value was 146 mM, and simulated limit value was 437 mM. For *A. pictum* seeds stored for 2 years, *y* = −0.1419*x* + 52.6466 (*R*^2^ = 92%), simulated critical value was 19 mM, and simulated limit value was 371 mM (Figure 6).

## Discussion

Some aspects of ecology, biology, and utilization in *A. venetum* and *A. pictum* have been extensively studied (Thevs et al., 2012; Rouzi et al., 2018; Jiang et al., 2019; Li et al., 2020), yet our data are the first to study the simultaneous effects of storage period and various abiotic factors on germination of both species. The germination niches of *A. venetum* and *A. pictum* are highly dependent on storage period, temperature regime, drought, and salinity. In addition, 1- or 2-year-stored seeds of *A. venetum* have higher drought tolerance than that of *A. pictum*. The results indicate that seed storage dramatically decreases drought and salinity tolerance during seed germination for both species, especially for seeds stored for 2 years.

Seed storage significantly decreased seed germination percentage and velocity of both *Apocynum* species, especially under stress conditions. In our experiment, germination percentage of freshly matured seeds of *A. pictum* was above 80% under suitable germination conditions and can tolerate 600 mM NaCl. However, germination percentage of seeds after 2-year storage decreased to 60%, and even decreased to 0% under 400 mM NaCl. Seeds of *A. venetum* also showed a decreased stress tolerance after storage. This should be related to the accumulation of ROS during the storage and under salinity and osmotic stress (Moncaleano-Escandon et al., 2013; Lozano-Isla et al., 2018). Oxidative stress is the major contributor to seed deterioration and the decrease of seed longevity. High ROS concentration causes lipid peroxidation and damages cell integrity (Groot et al., 2012; Zhou et al., 2019). Decrease in the germination of aged *A. venetum* seeds is related to the decrease in antioxidant enzyme activity and increase in malondialdehyde (MDA), suggesting a close relationship between the deterioration of biological membranes and the loss of germinability (Liu et al., 2015). Our results indicate that seeds of *A. venetum* and *A. pictum* stored at room temperature will gradually lose viability. Further studies are needed to determine optimum storage conditions for long-term storage.

Light did not significantly affect seed germination of *A. venetum* and *A. pictum*. The results indicate that seeds of both species do not require light or darkness to facilitate germination. According to the responses of seeds to light, there are three categories: (1) seeds that germinate only in the darkness, (2) seeds that germinate in continuous light or a brief amount of light, and (3) seeds that can germinate in light or darkness (El-Keblawy, 2017; Yan and Chen, 2020). Different types of seeds have different ecological adaptation. Generally, light requiring seeds tend to be very small (Koutsovoulou et al., 2014; Yan and Chen, 2020). In accordance with this general rule, seeds of *A. venetum* and *A. pictum* that are not very small do not have special need of light condition. Thus, in arid regions, seeds of both species should normally be sown at shallow depth to maintain proper humidity level and for effective seedling emergence.

Germination timing might be expected to coincide with periods when the soil salinity was diluted by rain, perhaps accompanied by cooler temperatures, which would reduce evaporation (Huang et al., 2003; El-Keblawy et al., 2018). Contrary to our expectation, though low and high temperature regimes delay germination, freshly matured seeds of both species can complete germination in 10 days at all test temperature regimes. For seeds stored for 1 or 2 years, optimum temperature regime for germination is 10/25°C. This indicates that interactive effects of seed storage and unsuitable temperatures are more severe. Similarly, seed storage aggravates the inhibitory effects of sub/supra-optimal temperatures (Rasool et al., 2017). Thus, for crop planting, freshly matured seeds can be sowed at a wide range of temperature; however, stored seeds can only be sowed at optimal temperature for successful seedling establishment. 10/25°C represents the mean daily maximum and minimum monthly temperatures of May in study area. In practice, we can sow seeds of both species in this month.

Plants living in arid regions are expected to experience high levels of drought stress (Cui et al., 2019; Rasheed et al., 2019). Our results indicate that seeds of *A. venetum* and *A. pictum* have high drought tolerance and show differential characteristics. Freshly matured seeds of *A. venetum* and *A. pictum* have similar drought tolerance. The simulated critical value (50% germination) for *A. venetum* seeds is 29.56% and the value for *A. pictum* is 26.58%. After 1 year of storage, seeds of *A. venetum* did not show significantly decrease in drought tolerance compared with freshly matured seeds. However, drought tolerance of *A. pictum* seeds stored for 1 year decreased dramatically. There is evidence that simulated limit values for *A. venetum* and *A. pictum* seeds stored for 1 year are 40.55 and 30.16%. After stored for 2 years, seeds of both species showed a different trend of decrease in drought tolerance. In short, after stored for 1 or 2 years, *A. venetum* seeds own higher drought tolerance than *A. pictum* seeds during germination stage. Germination performance of seeds of both *Apocynum* species decreased with the increase of storage period and drought stress. With the increase of PEG concentrations, MDA content of *A. venetum* seeds increases and activities of SOD and POD increase first and then decrease (Xu et al., 2015). Thus, the phenomenon that seed storage significantly decreases drought tolerance may be explained by the dual accumulation of MDA and decrease of SOD and POD activities of stored seeds under drought stress.

Plants of both *Apocynum* species living in arid regions are usually subjected to high salinity (Jiang et al., 2015). Although the adult plants of both species are well adapted to salinity, seed germination is inhibited by saline conditions (Shi et al., 2014). *Apocynum* seeds germinated to highest percentages in fresh water, and low salinity concentration (50–200 mM NaCl) did not significantly affect germination percentage of freshly matured seeds. Freshly matured seeds of both species have similar salt tolerance. The simulated limit value for freshly matured seeds of *A. venetum* seeds was 653 mM and the value for *A. pictum* was 631 mM. However, a rapid decrease in germination occurs for seeds of 1- or 2-year storage with the increase of salt concentration. Seeds of most halophytes can tolerate 250–800 mM NaCl during germination (Flowers and Colmer, 2008). However, seeds of a minority of halophytes are quite salt tolerant and may germinate in 1000–2000 mM NaCl (Gul et al., 2013). Thus, seeds of both species show middle-degree salt tolerance. Moreover, this study clearly indicates that seed germination of *A. venetum* and *A. pictum* is influenced not only by the osmotic potential (caused by salt concentration) but also by the ion toxicity. For example, 400 mM NaCl and 26% PEG-6000 have the equal osmotic potential (−1.5 MPa). Germination percentage of *A. venetum* seeds stored for 1 year was less than 3% at 400 mM NaCl; however, germination percentage was above 30% at 26% PEG-6000.

Both *Apocynum* species are suitable for rehabilitation of degraded saline soils in arid regions. First, seeds of both plants germinate quickly at the wide temperature regimes regardless of light conditions. This is important for seedling establishment in arid environments that confront severe water scarcity. Second, not only adult plants but also germinating seeds of both *Apocynum* species can tolerate high drought and salinity stresses. Third, propagation of *Apocynum* in the field mainly depends on the perennating buds on underground rhizome. Furthermore, we also find that it is easy for both species to establish in the first sowing in the field experiment. Compared with *A. venetum*, *A. pictum* adapts better to extremely arid conditions (Rouzi et al., 2018). Thus, *A. pictum* may be more suitable for artificial plantation in more water-stressed region.

## Conclusion

Taken together, our results indicate that light conditions do not significantly affect seed germination of *A. venetum* and *A. pictum*. Meanwhile, the optimal temperature regime for seed germination of both species is 10/25°C. Drought and salinity significantly decrease germination percentage and velocity, especially for seeds stored for more than 2 years. Thus, in agricultural practice, we’d better sow freshly matured seeds or seeds stored for 1 year in arid and semiarid zones. We can sow them at optimal temperature (10/25°C) under low salinity conditions in the shallow layer of lands using drip-irrigation. We are suggesting that *Apocynum* seeds stored for 2 years should not be used for the safety of agricultural production.

## Data Availability Statement

The raw data supporting the conclusions of this article will be made available by the authors, without undue reservation.

## Author Contributions

LJ, CT, and LW conceptualized and designed the study. CS and LJ collected the data. CS, MT, LJ, CT, and LW wrote the manuscript. CS, MT, LJ, CT, and LW reviewed the manuscript. All authors read and approved the final manuscript.

## Conflict of Interest

The authors declare that the research was conducted in the absence of any commercial or financial relationships that could be construed as a potential conflict of interest.
